# Supplementary material for: Quality of Tenebrio molitor Powders: Effects of Four Processes on Microbiological Quality and Physicochemical Factors
Source: Foods. 2023 Jan 28;12(3):572. doi: 10.3390/foods12030572 (PMC9914264; doi:10.3390/foods12030572)
Supplement: Supplementary file 1 [file foods-12-00572-s001.zip › foods-2133326-supplementary.pdf]

Appendix A:

Supplementary Materials

Table 1. Microbial counts of fresh *T. molitor* larvae in log CFU/g.

Sample	Batch	Total aerobic count (TAC)	Bacterial endospores	Lactic acid bacteria(LAB)	*Enterobacteriaceae*	Yeasts and molds	Yeast	Molds
	1	8.5±0.0	5.3±0.0	8.1±0.0	/	4.2	3.9*	3.9*
A	2	8.3±0.0	3.5±0.1	7.9±0.1	>4.2	3.8*	2.3	3.8*
	Mean	8.4±0.0	5.0±0.0	8.0±0.1	>4.2	4.0	3.1	3.8
	1	8.4±0.0	4.1±0.1	8.0±0.1	/	/	/	4.0*
AR	2	8.5±0.0	3.6±0.1	7.9±0.1	/	/	/	4.0*
	Mean	8.4±0.0	3.9±0.1	7.9±0.1	/	/	/	4.0

* Calculation performed on one dilution; / Not tested

Table 2. Microbial hazards detection of fresh *T. molitor* larvae in log CFU/g.

Sample	Batch	*E.coli*	Sulfite-reducing anaerobes	Coagulase-positive *staphylococci*	*Bacillus cereus*	*Clostridium perfringens*	*Listeria monocytogenes*	*Salmonella* spp.	*Cronobacter sakazakii*
	1	<1.0	1.0**	<2.0	<1.0	<1.0	ND	ND	ND
A	2	<1.0	<1.0	<2.0	<1.0	<1.0	ND	ND	ND
	Mean	<1.0	<1.0	<2.0	<1.0	<1.0	ND	ND	ND

** Estimated number; ND not detected

Table 3. Microbial counts of *T. molitor* powder in log CFU/g.

Sample	Batch	Total aerobic count (TAC)	Bacterial endospores	Lactic acid bacteria (LAB)	*Enterobacteriaceae*	Yeasts and molds	Yeast	Molds
PA-P	1	5.6±0.1	2.5±0.1	3.9±0.1	3.6*	3.4*	3.4*	1.5**
	2	4.6±0.1	2.2±0.1	3.2±0.0	2.0**	<1.0	<1.0	<1.0
	Mean	5.3±0.1	2.4±0.1	3.7±0.1	2.8	<2.2	<2.2	<2.2
PB-P	1	4.4±0.0	2.3±0.1	2.0±0.2*	<1.0	<1.0	<1.0	<1.0
	2	4.9±0.1	2.2±0.1	3.2±0.0	2.8	<1.0	<1.0	<1.0
	Mean	4.7±0.1	2.2±0.1	3.0±0.0	<1.9	<1.0	<1.0	<1.0
PC-P	1	5.5±0.1*	2.6±0.1	5.1±0.1	3.4*	<1.6	<1.0	1.5**
	2	5.5±0.1*	2.4±0.1	3.8±0.1	3.2	2.1	2.1	1.0**
	Mean	5.5±0.1	2.5±0.1	4.8±0.1	3.3	<1.9	<1.6	1.3
PD-P	1	8.5±0.0	5.2±0.0	7.8±0.1	>4.2	2.6	2.0**	2.5
	2	8.8±0.1	4.4±0.0	8.3±0.0	>4.2	2.9	2.5	2.7
	Mean	8.6±0.1	4.9±0.0	8.1±0.0	>4.2	2.8	2.3	2.6

* Calculation performed on one dilution; ** Estimated number

Table 4. Microbial hazards detection of *T. molitor* powder in log CFU/g.

Sample	Batch	*E.coli*	Sulfite-reducing anaerobes	Coagulase-positive *staphylococci*	*Bacillus cereus*	*Clostridium perfringens*	*Listeria monocytogenes*	*Salmonella* spp.	*Cronobacter sakazakii*
	1	<1.0	<1.0	<2.0	<1.0	<1.0	/	/	/
PA-P	2	<1.0	<1.0	<2.0	<1.0	<1.0	/	/	/
	Mean	<1.0	<1.0	<2.0	<1.0	<1.0	/	/	/
	1	<1.0	<1.0	<2.0	<1.0	<1.0	/	/	/
PB-P	2	<1.0	<1.0	<2.0	<1.0	<1.0	/	/	/
	Mean	<1.0	<1.0	<2.0	<1.0	<1.0	/	/	/
	1	<1.0	<1.0	<2.0	<1.0	<1.0	/	/	/
PC-P	2	<1.0	<1.0	<2.0	<1.0	<1.0	/	/	/
	Mean	<1.0	<1.0	<2.0	<1.0	<1.0	/	/	/
	1	<1.0	<1.0	<2.0	<1.0	<1.0	/	/	/
PD-P	2	<1.0	<1.0	<2.0	<1.0	<1.0	/	/	/
	Mean	<1.0	<1.0	<2.0	<1.0	<1.0	/	/	/

/ Not tested

Table 5. Log reduction of significant hazards estimated with D_{ref}-values for two heat treatments.

Log reduction	*B. Cereus (spore)*	*C. botulinum group I (spore)*	*C. perfringens (spore)*	*Cronobacte r* spp.	*E. coli STEC*	*L. monocytogenes*	*S. aureus*	*Salmonella* spp.	*S. aureus enterotoxin*
100°C for 5 min	6.28	0.18	0.12	15789721.53*	863245.78*	3799.78*	258973.73*	1187585.56*	0.03
80°C for 30 min	0.95	0.01	0.02	25415.41*	1199.48*	346.52*	2159.06*	5968.88*	0.05

* Replaced by >12 in Figure
